# Retraction Note: Exploring predictors and prevalence of postpartum depression among mothers: multinational study

**DOI:** 10.1186/s12889-026-29030-4

**Published:** 2026-08-17

**Authors:** Samar A. Amer, Nahla A. Zaitoun, Heba A. Abdelsalam, Abdallah Abbas, Mohamed Sh Ramadan, Hassan M. Ayal, Samaher Edhah Ahmed Ba-Gais, Nawal Mahboob Basha, Abdulrahman Allahham, Emmanuael Boateng Agyenim, Walid Amin Al-Shroby

**Affiliations:** 1 Department of Public Health and Community Medicine, Faculty of Medicine, Zagazig University, Zagazig, Egypt; 2 Department of Family Medicine, Faculty of Medicine, Zagazig University, Zagazig, Egypt; 3 Department of Psychiatry, Faculty of Medicine, Zagazig University, Zagazig, Egypt; 4 Faculty of Medicine, Al-Azhar University, Damietta, Egypt; 5 Department of Obstetrics and Gynecology, Faculty of Medicine, Zagazig University, Zagazig, Egypt; 6 Hammurabi Medical College, University of Babylon, Al-Diwaniyah, Iraq; 7 Hardamout University College of Medicine, Almukalla, Yemen; 8 Department of General Medicine, Shadan Institute of Medical Science, Hyderabad, India; 9 College of Medicine, Sulaiman Alrajhi University, Albukayriah, Al-Qassim, Saudi Arabia; 10 Department of Virology, Noguchi Memorial Institute for Medical Research, University of Ghana Legon, Accra, Ghana; 11 Department of Public Health and Community Medicine, Faculty of Medicine, Beni- Suef University, Beni-Suef, Egypt

**Retraction Note: BMC Public Health 24, 1308 (2024)**

**https://doi.org/10.1186/s12889-024-18502-0**

The Editor has retracted this article. Following publication, concerns were raised regarding the data reported in this article. During the investigation of these concerns, it was identified that the study does not appear to have ethics approval in all countries where it was carried out. The authors have been unable to provide satisfactory evidence to demonstrate that the study was conducted with the appropriate ethical oversight, and the Editor has therefore lost confidence in the study.

Author Samar A. Amer has indicated that all authors disagree with this retraction.

